# Selected Parameters Which Support the Laboratory Diagnosis of Cardiovascular-Kidney-Metabolic Syndrome in the Light of Current Guidelines: A Narrative Review

**DOI:** 10.3390/jcm15041456

**Published:** 2026-02-12

**Authors:** Michał Sławiński, Justyna Bugajska, Katarzyna Dąbrowska, Jacek Różański, Andrzej Brodkiewicz, Jeremy S. C. Clark, Violetta Sulżyc-Bielicka, Robert Nowak, Dorota Kostrzewa-Nowak

**Affiliations:** 1 Department of Clinical and Molecular Biochemistry, Pomeranian Medical University in Szczecin, 72 Powstańców Wlkp. Al., 70-111 Szczecin, Poland; michal.slawinski@pum.edu.pl (M.S.); jeremy.clark@pum.edu.pl (J.S.C.C.); 2 Department of Laboratory Diagnostics, Pomeranian Medical University in Szczecin, University Clinical Hospital, No. 2, 72 Powstańców Wlkp. Al., 70-111 Szczecin, Poland; j.bugajska@usk2.szczecin.pl (J.B.); k.dabrowska@usk2.szczecin.pl (K.D.); 3 Department of Nephrology, Transplantology and Internal Medicine, Pomeranian Medical University in Szczecin, 72 Powstańców Wlkp. Al., 70-111 Szczecin, Poland; jacekrozanski@wp.pl; 4 Department of Pediatrics, Pediatric Nephrology, Dialysis and Acute Intoxications, Pomeranian Medical University, 4 Mączna St., 70-204 Szczecin, Poland; a.brodkiewicz@gmail.com; 5 Department of Clinical Oncology, Pomeranian Medical University, 4 Arkońska St., 71-455 Szczecin, Poland; violettasulzycbielicka@gmail.com; 6 Institute of Physical Culture Sciences, University of Szczecin, 17C Narutowicza St., 70-240 Szczecin, Poland; robert.nowak@usz.edu.pl; 7 Department of Pathology, Pomeranian Medical University in Szczecin, 1 Unii Lubelskiej St., 71-242 Szczecin, Poland

**Keywords:** cardiovascular-kidney-metabolic syndrome, kidney disease, laboratory diagnostic

## Abstract

Progression in understanding the relationships among cardiovascular, kidney, and metabolic diseases necessitates reappraising these concepts. Here, this narrative review explains the evolution of the ideas behind cardiovascular-kidney-metabolic syndrome (CKMS), focusing both on the impact of kidney disease on the cardiovascular system and metabolic syndrome and, conversely, on the effects of metabolic syndrome on cardiovascular and kidney diseases. Merging these concepts has resulted in a holistic approach more pertinent to managing the increased pressure from civilization diseases. In light of recent guidelines, early laboratory assessment is critical for risk stratification by improved patient classification, enabling individualized therapeutic strategies. Moreover, understanding the molecular mechanisms common to these systemic disorders not only enhances diagnostic accuracy but also facilitates the implementation of preventive measures that target multiple organ pathologies simultaneously. This review summarizes selected laboratory parameters that may support the diagnosis and management of cardiovascular-kidney-metabolic syndrome, aligning current knowledge with emerging clinical recommendations.

## 1. Introduction and Definitions

Metabolic syndrome (MS) is an important research topic in both diabetology and cardiology as it is associated with the occurrence of specific cardiovascular risk factors such as dyslipidemia, insulin resistance, hypertension, visceral obesity, and carbohydrate metabolism disorders. As knowledge has advanced, the inextricable links between these and the cardiovascular and renal systems have been emphasized.

**Figure 1:**
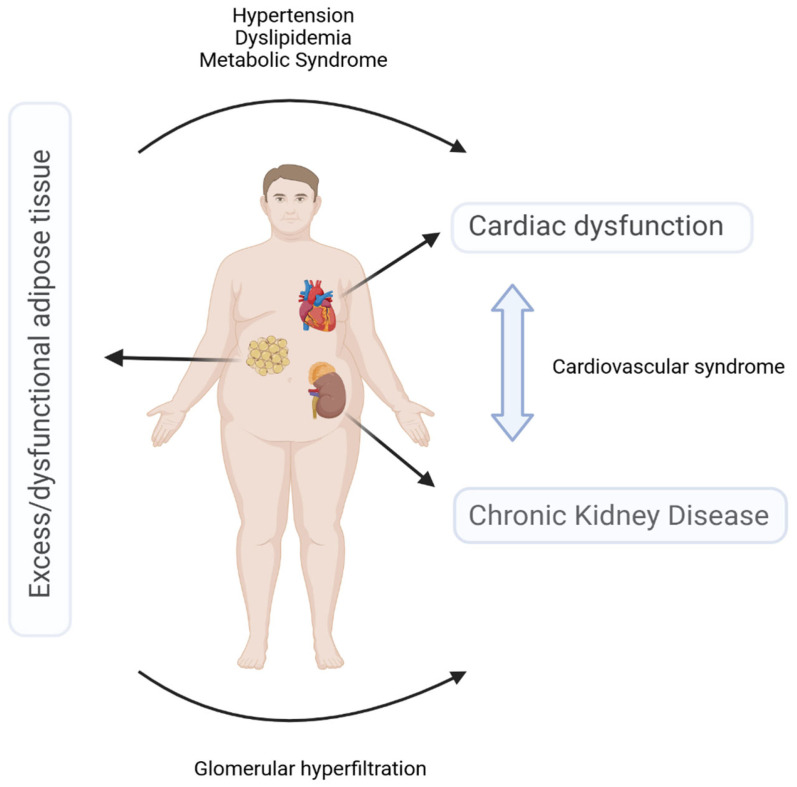
The multidisciplinary approach to cardiovascular-kidney-metabolic syndrome. Created in BioRender by Sławiński, M. (2025; https://biorender.com/dnytndw, accessed on 16 October 2025).

Due to the significant prevalence of coexisting kidney, cardiovascular, and metabolic disorders, the American Heart Association (AHA) defined cardiovascular-kidney-metabolic syndrome (CKMS; otherwise known as cardiorenal-metabolic syndrome and sometimes loosely referred to as cardiovascular-metabolic syndrome) as a distinct disease entity in 2023. This syndrome has been described as “a systemic disorder characterized by pathophysiological interactions between metabolic risk factors, chronic kidney disease (CKD), and the cardiovascular system, which ultimately leads to multi-organ dysfunction and a high risk of serious cardiovascular complications” [1]. As the patients’ understanding of the disease is critical, the definition was subsequently simplified to: “a health disorder resulting from the links between heart disease, kidney disease, and metabolic disorders leading to adverse health outcomes” [2]. The relationship with, and potential development from, cardiorenal syndrome is described in Braunwald [3]; however, here we focus on the possible development from excess or dysfunctional adiposity, as well as a renal component, as a new aspect of the recent understanding of CKMS. Defining the concept of CKMS opened the door to implementing an interdisciplinary, long-term approach to risk assessment, early prevention, and the treatment of the vicious cycle created by overlapping cardiovascular, renal, and metabolic risk factors. These form a pathological feedback loop between heart disease, CKD, and type 2 diabetes, and indicate dysfunctional adipose tissue as the initiating factor (Figure 1) [4,5,6].

Classical MS focuses on risk factors that include visceral obesity, lipid abnormalities, hypertension, and insulin resistance. MS identifies individuals at increased cardiometabolic risk, but it has limited insight into organ damage, particularly to the kidneys and heart [7]. Alongside the development of the MS concept, increased attention was paid to the close relationship between the kidneys and the heart, leading to the definition of cardiorenal syndrome (CRS). This term emphasizes the possible coexistence of cardiac and renal dysfunction and the impact of dysfunction in one organ on the other. The mechanism of CRS is complex and still not fully understood, and one aspect of the syndrome’s development is assumed to be based on the hemodynamic disturbances associated with heart failure [8]. However, the definition of CRS does not adequately account for primary metabolic factors that may also initiate dysfunction of the heart and kidneys.

The CKMS definition unifies these areas by indicating that the metabolic basis of specific disorders, such as hyperglycemia, oxidative stress, and chronic inflammation, could be a common factor leading to the progression of both heart and kidney disease [9].

Conventional therapeutic strategies have focused on the isolated treatment of specific disorders, such as diabetes, hypertension, and dyslipidemia. The conception of CKMS, however, justifies a holistic approach to therapy, encompassing both lifestyle modifications and pharmacological treatment, aimed at simultaneously minimizing metabolic, cardiovascular, and renal risk [5].

The purpose of this paper is to discuss selected laboratory parameters that can support the diagnosis of cardiovascular-kidney-metabolic syndrome.

## 2. An Overview of the Pathomechanism of CKMS

**Figure 2:**
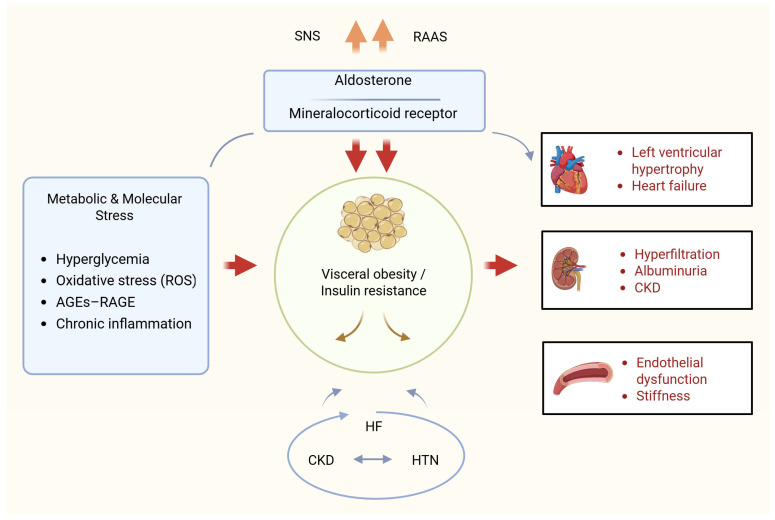
The pathomechanism of cardiovascular-kidney-metabolic syndrome. Created in BioRender by Sławiński, M. (2026; https://BioRender.com/j4pf4l5, accessed on 21 January 2026). AGEs, advanced glycation end products; CKD, chronic kidney disease; HF, heart failure; HTN, hypertension; RAAS, renin-angiotensin-aldosterone system; RAGE, receptor for advanced glycation end products; ROS, reactive oxygen species; SNS, sympathetic nervous system.

The pathophysiological consequences of CKMS reflect multifaceted relationships between metabolic risk factors and diseases that affect the heart and kidneys (Figure 2) [10,11].

The pathophysiology of CKMS is driven, among other factors, by sympathetic nervous system overactivity, the renin-angiotensin-aldosterone system (RAAS), and a range of hemodynamic and neurohormonal mediators, including nitric oxide, prostaglandins, and oxidative stress. The molecular basis of the disease is closely linked to glycemic disorders, which increase the synthesis of reactive oxygen species (ROS) [9,11,12]. Increasing oxidative stress is one of the primary sources of fibrosis and thus, through several pathways—including activation of the polyol and hexosamine pathways, which exacerbate oxidative stress in the cellular cycle—activation of protein kinase C (PKC), formation of glycation end-products (AGEs) triggered by non-enzymatic glycation and upregulation of their receptor RAGE [9,11,13]. The literature data indicate that AGEs can directly damage the heart, blood vessels, and kidneys. AGE-related mechanisms may contribute to the risks associated with diabetes, including diabetic cardiomyopathy, diabetic dermatitis (in CKD), and aggressive diabetes. AGEs and ROS are also associated with endothelial dysfunction, and this dysfunction significantly contributes to the occurrence of microvascular and macrovascular complications [9,11,13]. It is also known that local activation of the RAAS in the myocardium and kidneys may be a consequence of glycemic control systems, among others, promoting vasoconstriction, fibrosis, and exacerbation of organ signaling [9,11,13,14]. The cytopathology associated with CKMS suggests that endoplasmic reticulum (ER) stress, calcium disorders, oxidative stress, and chronic inflammation underlie these processes [15]. It is important to note that excessive dietary intake of high-fat and high-glycemic foods correlates with the development of insulin resistance [16]. This occurs through activation of the mTOR/S6 kinase 1 signaling pathway in cardiovascular and renal tissue, thus contributing to the complex association with CKMS [16,17].

CKMS is most frequently triggered by excessive and dysfunctional adipose tissue, which contributes to decreased insulin sensitivity. With its concomitant pro-inflammatory and pro-oxidant properties, visceral adipose tissue plays a crucial role in this process. Ongoing inflammatory processes reduce tissue sensitivity to insulin. Insulin resistance leads to impaired glucose tolerance, which results in hyperglycemia and induces glomerular hyperfiltration, also contributing to the development of nonalcoholic fatty liver disease. Activation of these mechanisms initially attempts to compensate for metabolic disturbances, but in the long term, it contributes to the development of CKD and primary hypertension. Due to peripheral vascular damage, these further contribute to the impairment of myocardial and renal perfusion and consequently, overlapping secondary hypertension. Hypertension, in turn, promotes left ventricular hypertrophy leading to the development of heart failure (HF) [1,5,9,18].

Patients with hypertension also experience hormonal disorders (endocrine dysregulation) related to, among others, excessive aldosterone activity, which not only regulates water and electrolyte balance but also influences prooxidant, proinflammatory, and pro-fibrotic effects in vessels, kidneys, and the heart, promoting the development of hypertension, renal dysfunction, and cardiovascular disease in patients with CKMS [19]. Blood aldosterone concentration significantly correlates with the presence of vascular changes, suggesting that hormonal dysregulation is closely linked to the progression of cardiovascular changes.

Epidemiological studies show that hypertension is prevalent in patients with CKD and is an independent predictor of renal function deterioration and cardiovascular events, which also highlights the importance of hypertension in the pathogenesis of CKMS [20,21]. Clinical studies have found that hypertension often remains poorly controlled in the CKD population, despite the availability of effective therapeutic strategies, which is associated with a higher risk of renal disease progression and worse cardiac outcomes [20]. High blood pressure causes damage to the renal vessels and renal parenchyma through hemodynamic mechanisms and stimulation of the RAAS, which contributes to hypertensive nephropathy with predominant vascular damage and a further decline in glomerular filtration [21]. Metabolic disorders, including elevated blood pressure, are strongly associated with the development and progression of CKD in the context of CKMS, and the accumulation of these factors increases the likelihood of kidney damage [22,23]. Patients with hypertension and additional criteria for CKMS have a significantly higher risk of CKD than those without metabolic disorders, confirming the synergistic effect of hypertension with other metabolic risk factors [23]. Early-onset hypertension correlates with a higher risk of developing CKD, which may have important implications for risk stratification in CKMS [24]. Because of these associations, adequate blood pressure control is fundamental not only for limiting CKD progression but also for reducing cardiovascular burden in CKMS. Integrating hypertension as a central component in both research and clinical care of patients with CKMS is therefore crucial to improving health outcomes in this population.

Chronically elevated aldosterone levels lead to sustained activation of the mineralocorticoid receptors in renal tubular cells, podocytes, and mesangial cells, which initiates a cascade of renal damage. Activation also promotes increased oxidative stress and increased expression of proinflammatory cytokines [25]. This, in turn, leads to increased sodium retention, increased intraglomerular pressure, and progressive albuminuria, which are strong predictors of accelerated renal dysfunction [26]. Long-term exposure to aldosterone also induces fibrotic processes through fibroblast activation, increased collagen synthesis, and stimulation of TGF-β pathways, which promotes irreversible damage to the renal parenchyma [26]. This confirms that aldosterone is a key mediator linking metabolic, cardiac, and renal disorders. Consideration of the pathogenic mechanisms is crucial for risk stratification and selecting therapy aimed at slowing the progression of CKD within the cardiorenal-metabolic syndrome.

Data from the literature also indicate that the course and severity of disorders in CKMS are sex-dependent. Hormonal disorders also influence sex differences in the course and severity of CKMS, which is modulated by the effects of sex hormones. Estrogens and testosterone modulate the circulatory system, glucose metabolism, and renal function [27]. Estrogens exert a protective effect by improving endothelial function, modulating the inflammatory response, and increasing insulin sensitivity, thereby delaying the progression of cardiorenal-metabolic disorders in premenopausal women [27,28]. In turn, estrogen deficiency in the postmenopausal period is associated with increased insulin resistance, increased blood pressure, and accelerated progression of CKD [28]. In men, androgen axis disorders, including reduced testosterone levels, correlate with an increased risk of visceral obesity, metabolic dysfunction, and adverse cardiovascular events, which further emphasizes the importance of considering hormonal differences in the risk assessment and personalization of therapeutic strategies in CKMS [29].

The presence of ectopic adipose tissue, particularly in the epicardium and pericardium, can lead to organ damage and exacerbate myocardial dysfunction, thereby increasing the risk of arrhythmias such as atrial fibrillation [1,5,30].

As mentioned above, the reverse relationship also exists: heart failure, through reduced renal perfusion, can lead to deterioration of renal function as evidenced by a decrease in the estimated glomerular filtration rate (eGFR). This mechanism involves diminished cardiac output, increased venous pressure, and activation of the RAAS and the sympathetic nervous system.

The mechanisms underlying the mutual, often bidirectional, interactions among heart disease, CKD, and diabetes are increasingly well-defined. Even so, as emphasized by the American Heart Association (AHA), knowledge in this area remains incomplete [1,4,5].

## 3. Epidemiological Data and Classification

In 2021, 43 million people worldwide died due to recorded noncommunicable diseases (including 18 million under the age of 70) from an estimated total number of deaths of 68 million. Data from the Institute for Health Metrics and Evaluation show that cardiovascular diseases (19 million) accounted for the largest fraction of deaths, followed by cancer (10 million), chronic respiratory diseases (4 million), and diabetes, including diabetic kidney disease (2 million). The main modifiable risk factor for cardiovascular disease was hypertension, which caused approximately 10.4 million deaths annually [31].

The occurrence of cardiovascular-kidney-metabolic syndrome affects a large proportion of the population, both in Poland (relevant Polish guidelines are discussed below) and worldwide. In 2025, it was estimated that diabetes, obesity, and other components of the metabolic syndrome affected over a billion people worldwide [5].

In Poland a continuous increase in cases of hypertension (>10 million people); type 2 diabetes (>3 million); overweight (>15 million); obesity (>4 million); lipid disorders coexisting with metabolic syndrome (>3 million); ischemic heart disease (>2 million); or CKD (>700,000 registered, but according to some experts affecting ~4 million people) has been observed. This has forced the development of novel strategies to address an aging society [2].

Early detection of metabolic syndrome components offers the potential to prevent its adverse consequences, especially given the availability of effective therapies. Proactive screening, conducted both in the general population and in clinical settings, is a key element of CKMS diagnosis, enabling the identification of individuals at various stages of its development [32].

According to AHA recommendations, there are two major screening strategies: addressing biological factors indicating kidney function or cardiovascular disease (CVD); and addressing so-called Social Determinants of Health (SDOH), which consider social components and potential barriers that support or challenge healthy lifestyles [5]. According to the WHO, the most essential SDOH include: income and economic status; education; employment and working conditions; housing and environmental conditions; access to healthcare; access to healthy food; social integration and lack of discrimination; social safety and stability; and early childhood development [33].

MS has been defined by various global organizations, such as: the World Health Organization (WHO); the European Group for the Study of Insulin Resistance (EGIR); the American Association of Clinical Endocrinology (AACE); the American Heart Association/National Heart, Lung, and Blood Institute (AHA/NHLBI); the International Diabetes Federation (IDF); and the National Cholesterol Education Program Adult Treatment Panel III (NCEP-ATPIII).

The established diagnostic criteria, developed by these six organizations, along with example parameters, are shown in Table 1.

The presence of at least 3 of the five criteria listed in Table 1 is required for diagnosis [32,35]. For three organizations (EGIR, WHO, AACE), one of these must be insulin resistance or impaired glucose tolerance. Furthermore, inclusion of pharmacological treatment for any of the five key metabolic parameters is crucial for diagnosing metabolic syndrome in some patients: pharmacological treatment concerning a criterion is considered equivalent to that criterion. This means that patients who use pharmacological therapy to control obesity, insulin resistance, lipid abnormalities, hypertension, and/or hyperglycemia can be diagnosed with metabolic syndrome even if the values of their parameters are within normal ranges. This approach reflects the contemporary understanding of metabolic syndrome as a complex disorder in which the need for therapeutic intervention itself is a significant risk indicator [36].

In 2022, the diagnostic criteria for MS were established in Poland (Table 1) and are characterized by obesity, with two of the following three: impaired glucose metabolism, elevated non-high-density-lipoprotein (HDL) cholesterol (atherogenic dyslipidemia), or elevated blood pressure [34]. These were developed by Polish national medical societies including the Polish Society of Arterial Hypertension (PTNT), the Polish Society of Parenteral, Enteral and Metabolism Nutrition (POLSPEN), the Polish Society for the Treatment of Obesity (PTLO) and the Polish Society of Family Medicine (PTMR); as a joint position statement taking into account local epidemiological data and clinical practice. Although these broadly reflect the international background, two significant differences are present:

1. Obesity is a mandatory criterion, meaning it must be present for the diagnosis to be considered.
2. The number of additional criteria is limited to three, of which two must be met: impaired glucose metabolism, elevated non-HDL cholesterol (atherogenic dyslipidemia), or elevated blood pressure.

The requirement of obesity as a mandatory criterion highlights the central role of excess adipose tissue as a key driver of metabolic dysfunction. The creation of independent but related criteria may be justified by the relatively high ethnic homogeneity of the Polish population, cultural and dietary specificities, and the realities of the Polish healthcare system, including the availability of laboratory tests and therapeutic options. The simplified structure of the criteria may facilitate their implementation in primary care settings, making these potentially efficient screening tools. On the other hand, simplicity challenges proper profiling and individualized therapeutic approaches.

The diagnostic criteria for MS, in addition to hypertension, include parameters of impaired glucose tolerance (see Table 1), and diabetes is also an independent risk factor for the development of CKD. The diagnostic criteria for diabetes and hypertension, as outlined in Polish and international recommendations, are presented in Table 2 below.

## 4. The Relationship Between Kidney Function and Metabolic Syndrome

Currently available data demonstrate a bidirectional association between metabolic syndrome and the development or progression of CKD [39]. Initial screening for CKD can significantly improve prognosis in patients at early stages of the disease. Still, on the other hand, the early stages of CKD are often asymptomatic and can easily be missed. Increasing screening frequency is therefore crucial for early detection and treatment of both CKD and CKMS [21].

CKD, according to the Kidney Disease: Improving Global Outcomes (KDIGO) guidelines, is defined as the presence of abnormalities in kidney structure or function that persist for at least 3 months, with either:

(1) Markers of kidney damage, regardless of eGFR, confirmed by:
  - albuminuria (albumin-to-creatinine ratio: ≥30 mg/g)
  - abnormalities in urine sediment (hematuria, erythrocyte casts)
  - structural abnormalities on imaging
  - history of kidney transplantation

or:

(2) Reduced glomerular filtration rate (eGFR) < 60 mL/min/1.73 m^2^

In clinically questionable situations, the KDIGO updated guidelines recommend using the CKD-EPI creat/CysC formula, based on creatinine and cystatin C levels, to diagnose CKD, particularly when creatinine and cystatin C values are low.

In 2010, the first recommendations for classifying cardiorenal-metabolic syndrome were published. Five subtypes of the syndrome were identified based on the primary organ involved and whether the disease is acute or chronic [30]. The steadily increasing incidence of the disease, affecting a considerable portion of the population, and resulting variability, later necessitated a broadening of the definition (shown in Table 3) [4,40].

Based on the above, initiating CKMS screening is recommended early in life:

(1) For young adults < 21 years of age:
  - Annual overweight/obesity screening using the Centers for Disease Control and Prevention growth percentile charts,
  - Blood pressure measurement starting at age 3 (in children without risk factors), at each visit if risk factors are present (overweight, diabetes, CKD, heart defects),
  - Mental and behavioral health: screening for adverse social factors that influence health for all children,
  - Fasting lipid panel between ages 9 to 11 and 17 to 21 (exception: age 2 if there is a family history of early CVD or familial hypercholesterolemia); In Poland, the lipid panel was included in the six-year assessment in the draft amendment to the regulation of the Minister of Health amending the regulation on guaranteed services in the scope of primary health care, which will come into force on 1 May 2025 [41].
  - Plasma glucose level/oral glucose tolerance test (OGTT) or glycated hemoglobin and other metabolic biomarkers from 9–11 years of age.
(2) In adults (≥21 years), in addition to the previously mentioned tests:
  - Screening for unfavorable social factors affecting health
  - Annual measurement of body mass index (BMI) and waist circumference
  - Screening for MS: hypertension, hypertriglyceridemia, low HDL cholesterol, hyperglycemia:
  - Detailed testing for liver fibrosis every 1–2 years in the presence of diabetes, prediabetes, or two metabolic risk factors
  - Assessment of coronary artery calcification in individuals with an average 10-year cardiovascular risk
  - Urine microalbuminuria albumin-to-creatinine ratio (µACR) and serum creatinine/cystatin C to assess CKD stage:
    - Annually for stage 2 CKM or higher
    - More frequently with higher KDIGO risk
  - Testing for subclinical heart failure (echocardiography and/or cardiac biomarkers) [1,40,42,43].

Although cardiovascular and metabolic parameters are often considered overlapping risk factors in the clinical approach, including kidney function adds remarkable value. In fact, the late stages of CKD have been considered as independent diagnostic parameters for advanced CKMS. However, the screening strategy involves kidney function assessment at early life stages and at the early stages of diagnosed CKMS, which represents a step forward in approaching the complexity of the disease. Remarkably, the renal screening involves both albuminuria and eGFR, which acts in accordance with the understanding of the pathomechanism of CKMS: initiated by hyperglycaemia and glomerular hyperfiltration as described above. Hence, this encompasses the possibility of early screening. However, little is known about the efficacy of specific therapeutic interventions at the early stages of the disease, and this requires further, possibly extensive, cohort investigations.

Additionally, one biomarker that could enhance the effectiveness of CKMS screening is serum uric acid (SUA). Uric acid is a purine byproduct eliminated by the kidneys [44]. Hyperuricemia is strongly associated with the development of cardiovascular disease, CKD, and metabolic disorders, including type 2 diabetes and hypertriglyceridemia. It often accompanies atherosclerosis, obesity, and hypertension [45]. It is also associated with endothelial abnormalities and decreased endothelial nitric oxide synthase (eNOS) activity, which, in turn, increases insulin resistance and reduces vasodilation [18]. Hyperuricemia is considered an independent risk factor for all components of CKD and therefore CKMS [46,47]. Studies have indicated a strong association between hyperuricemia and increased cardiovascular mortality, including myocardial infarction, heart failure, and stroke, possibly modified by the sympathetic nervous system [48]. In renal injury, SUA may be associated with endothelial dysfunction, oxidative stress, and overactivation of the RAAS [46].

## 5. Medical Laboratory Procedures in Cardiovascular, Kidney, and Metabolic Syndrome

Diagnostic methods allow the identification of individual components of the metabolic syndrome and the assessment of the risk of complications. Their proper application enables early detection of metabolic disorders, which is crucial for appropriate treatment and the prevention of cardiovascular disease and diabetes. It should be noted, however, that although the diagnostic criteria have been previously described, each of them has certain drawbacks and limitations, summarized in Table 4.

### Key Markers and Risk Stratification in CKMS

In everyday clinical practice, it is crucial not only to recognize CKMS but also to identify prognostic markers that can support risk stratification and therapeutic decisions. Effective diagnosis of CKMS requires a shift from simple disease detection to dynamic risk stratification. Basic parameters such as glucose and lipids remain essential, but the American Heart Association (AHA) guidelines include “key indicators” that help predict adverse outcomes:

- Renal function (eGFR and cystatin C) with eGFR based on creatinine is a standard. Even so, it has limitations in patients with reduced muscle mass or obesity (changes often present in individuals with CKMS). Combining cystatin C with eGFR (eGFRcr-cys) equations improves prognostic value for predicting cardiovascular death and renal failure,
- Albuminuria: The urinary albumin/creatinine ratio (UACR) is an independent predictor of cardiovascular disease. In CKMS, it reflects glomerular damage and serves as a surrogate marker of systemic endothelial dysfunction.
- Cardiac biomarkers: N-terminal pro B-type natriuretic peptide (NT-pro-BNP) and high-sensitivity troponin C (hs-cTn).

According to the AHA, NT-pro-BNP and hs-cTn play a key role in stage 3 CKMS (with subclinical cardiovascular disease). Elevated NT-proBNP and hs-cTn concentrations in asymptomatic patients with metabolic risk factors allow a reclassification of stage 1 disease into a higher-risk group, enabling faster implementation of aggressive cardioprotective therapy, e.g., SGLT2 inhibitors, to stop disease progression to heart failure [1,40]. Table 5 presents a comparison of diagnostic guidelines for patients with CKD based on the current KDIGO, European Society of Cardiology/European Association for the Study of Diabetes, and American Diabetes Association indications for the diagnosis and approach to CKMS.

Monitoring these three components—renal function (eGFR), renal damage (UACR), and cardiac stress (NT-pro-BNP)—might well result in the gold standard for the long-term assessment of patients with CKMS. Incorporating these markers into routine clinical practice, especially in conjunction with evaluation of metabolic factors (e.g., visceral obesity, glycemia, dyslipidemia), enables more precise risk stratification of renal disease progression and cardiovascular complications and can support decisions to intensify nephroprotective and cardioprotective therapy.

When assessing the obtained marker values, it is also reasonable to compare them with clinical recommendations for determining the risk of individual CKMS components, e.g., the KDIGO guidelines. Incorporating the KDIGO risk grid into the clinical approach to CKMS allows for objective risk stratification of CKD progression, which is essential for therapeutic decision-making and patient monitoring. The KDIGO guidelines classify kidney disease based on eGFR and albuminuria (UACR), which serve as the basis for assessing progression risk from low to very high, regardless of CKD etiology [49]. The literature data indicate that albuminuria combined with a reduced eGFR is strongly associated with a higher risk of major cardiovascular events, heart failure, and all-cause mortality, confirming the prognostic value of the KDIGO grid in assessing the clinical risk of patients with CKD [50]. Furthermore, results from studies such as SPRINT indicate that specific combinations of eGFR and albuminuria values according to the KDIGO classification are associated with different clinical risk profiles—for example, an increased risk of cardiovascular complications and death with albuminuria ≥ 30 mg/g, even with a moderate decrease in eGFR [51]. Combining these two indicators, the KDIGO risk grid allows not only to determine the risk of CKD progression but also to identify patients at high cardiometabolic risk, with direct implications for the frequency of blood pressure and glycemic control and the use of nephroprotective therapy. Monitoring changes in eGFR and UACR within the KDIGO risk grid can also support the assessment of disease progression. Integrating the KDIGO framework with individual patient metabolic and clinical markers enables more precise risk stratification and personalized treatment of CKMS, consistent with modern trends in the care of patients at high renal, cardiometabolic, and/or metabolic risk. Finally, using the risk grid as a prognostic tool is also important from a patient education perspective—patients informed about their current risk according to KDIGO demonstrate greater engagement in blood pressure control, dietary management, and lifestyle modification, which may delay CKD progression and reduce the burden of clinical complications.

## 6. Conclusions

As medical knowledge evolves, there is increasing evidence on the interrelationship between cardiovascular diseases, CKD, and metabolic disorders. Here, we have described criteria for diabetes, hypertension, CKD, and MS. Perhaps, in the future, if not already, the definition of MS will be subsumed into the broader determination of CKMS.

Many guidelines on cardiovascular prevention emphasize the importance of screening for hypertension, diabetes, and lipid disorders, which are among the main risk factors for developing cardiovascular-kidney-metabolic syndrome. Appropriate assessment and early modification of risk factors are, therefore, key elements in preventing this syndrome. Currently available data indicate that testing for CKMS risk factors should be most frequently performed in populations with established metabolic risk factors, such as CKD, diabetes, or hypertension. Avoiding excessive weight gain, which often occurs with age, will reduce the likelihood of developing CKMS risk factors and prevent the progression to subsequent stages of the syndrome. Therefore, including early markers of kidney injury appears complementary to previous approaches. For patients at stage “0”, the key elements are education, regular physical activity, a healthy diet, and avoidance of stimulants, possibly implemented within the whole population. Note that there are already screening strategies dedicated to early life stages, which include early kidney injury markers. Still, there is a need to maintain a balance between the simplicity of screening strategies and the complexity of individualized therapy, and, therefore, it is reasonable to assume that current guidelines provide a starting point for further clinical research. Properly designed endpoints that involve quality-of-life parameters should assess the efficacy of screening strategies, the interventions implemented, and, most importantly, the stratified individual approaches.

## Figures and Tables

**Table 1:** (**A**) Internationally established diagnostic criteria for metabolic syndrome according to six organizations * and the presence of some example parameters, and (**B**) criteria for the diagnosis of metabolic syndrome according to Polish guidelines from 2022 [32,34,35]. In (**A**), the presence of at least three of the five criteria is required for diagnosis. For three organizations (EGIR, WHO, AACE), one of these must be insulin resistance or impaired glucose tolerance. Pharmacological treatment can replace any parameter. In (**B**), two of the three parameter categories (A, B, or C) must be present.

(**A**) Internationally established diagnostic criteria	
Criterion	Example parameters
1. Obesity: elevated waist circumference or BMI	Waist circumference ≥ 88 cm for women and ≥102 cm for men (or if of Asian descent: ≥80 cm for women, ≥90 cm for men). BMI ≥ 30 kg/ m^2^
2. Insulin resistance or impaired glucose tolerance 3. Dyslipidemia (triglycerides) 4. Dyslipidemia (HDLC) 5. Hypertension	Glucose level ≥ 100 g/dL. Triglycerides ≥ 150 mg/dL. HDLC < 50 mg/dL for women, <40 g/dL for men. Systolic ≥ 130 mm Hg and/or diastolic ≥ 80 mm Hg and/or use of antihypertensive drugs.
(**B**) Criteria according to Polish guidelines	
Basic criterion	Abdominal obesity: waist circumference ≥ 88 cm (Females), ≥102 cm (Males) or BMI ≥ 30 kg/m^2^
Additional criteria	Aa. Fasting glucose ≥ 100 mg/dL or impaired glucose tolerance ≥ 140 mg/dL after 120 min of OGTT or Ab. Glycated hemoglobin ≥ 5.7% or Hypoglycemia treatment B. Non-HDL cholesterol ≥ 130 mg/dL or lipid-lowering treatment Ca. Blood pressure ≥ 130 mmHg systolic and/or ≥85 mmHg diastolic (office measurement), or Cb. Blood pressure ≥ 130 mmHg systolic and/or ≥80 mmHg diastolic (home measurement), or Cc. Antihypertensive treatment

OGTT, oral glucose tolerance test; HDL(C), high-density lipoprotein (cholesterol); BMI, body mass index. * the World Health Organization (WHO); the European Group for the Study of Insulin Resistance (EGIR); the American Association of Clinical Endocrinology (AACE); the American Heart Association/National Heart, Lung, and Blood Institute; the International Diabetes Federation; and the National Cholesterol Education Program Adult Treatment Panel III.

**Table 2:** Diagnostic criteria for diabetes and hypertension according to Polish and international recommendations [35,37,38].

	Source of Criteria	Criteria Parameters
Diagnosis of diabetes	American Diabetes Association (ADA)	HbA1c ≥ 6.5% (standardized methods according to NGSP) Fasting glucose ≥ 126 mg/dL (7 mmol/L) Glucose after 120 min of OGTT ≥ 200 mg/dL (11.1 mmol/L), test according to WHO standards In a person with symptoms of hyperglycemia or hyperglycemic crisis: random glucose ≥ 200 mg/dL (11.1 mmol/L)
	Polish Diabetes Association	Fasting blood glucose ≥ 126 mg/dL (7 mmol/L) on 2 different days Random blood glucose ≥ 200 mg/dL (11.1 mmol/L) with classic symptoms Blood glucose after 120 min of OGTT ≥ 200 mg/dL (11.1 mmol/L) HbA1c ≥ 6.5% (do not use as a criterion with anemia)
Diagnosis of hypertension	European Society of Cardiology (ESC)	Elevated blood pressure: 120/70 mmHg to <140/90 mmHg systolic/diastolic Hypertension: ≥140/90 mmHg systolic/diastolic Stage 2 hypertension: ≥180 mmHg systolic and/or ≥110 mmHg diastolic
	Polish Society of Arterial Hypertension/Polish Cardiology Society	Stage 1 hypertension: ≥140 mmHg systolic and/or ≥90 mmHg diastolic Stage 2 hypertension: ≥160 mmHg systolic and/or ≥100 mmHg diastolic Stage 3 hypertension: ≥180 mmHg systolic and/or ≥110 mmHg diastolic

NGSP, National Glycohemoglobin Standardization Program; OGTT, oral glucose tolerance test; WHO, World Health Organization.

**Table 3:** Developmental stages and diagnostic criteria for cardiovascular-kidney-metabolic syndrome according to the American Heart Association [1,4,40].

Stage	Description	Goal	Key Diagnostic Indicators
Stage 0	No risk factors.	Prevention, maintaining good cardiovascular health.	
Stage 1	Individuals with overweight/abdominal obesity or dysfunctional adipose tissue; no other metabolic risk factors	Identification and treatment of excess/dysfunctional adipose tissue	BMI ≥ 25 kg/m^2^ Waist circumference: men ≥ 102 cm, women ≥ 88 cm Fasting glucose ≥ 100 mg/dL < 125 mg/dL or HbA1c (glycated hemoglobin) ≥ 5.7–6.4%
Stage 2	People with metabolic risk factors or CKD	Lifestyle changes, treatment of modifiable risk factors	Hypertriglyceridemia ≥ 135 mg/dL HDLC: men < 40 mg/dL, women < 50 mg/dL Systolic blood pressure ≥ 130 mmHg or diastolic blood pressure ≥ 85 mmHg Fasting glucose ≥ 125 mg/dL
Stage 3	Subclinical atherosclerotic cardiovascular disease or subclinical heart failure in individuals with excess/ dysfunctional adipose tissue, other metabolic risk factors, or CKD	Delay or halt the progression of clinical disease with preventive measures	(i) Subclinical atherosclerotic cardiovascular disease—diagnosed based on the presence of coronary artery calcifications (coronary angiography or computed tomography angiography) (ii) Subclinical HF diagnosed based on elevated cardiac markers: NT-proBNP > 125 pg/mL High sensitivity Troponin T ≥ 14 ng/L for women, ≥22 ng/L for men High sensitivity Troponin I ≥ 10 ng/L for women, ≥12 ng/L for men (iii) Risk factors that burden the patient to the same extent as subclinical HF: CKD stage 4 (eGFR 15–29 mL/min/1.73 m^2^) or stage 5 eGFR < 15 mL/min/1.73 m^2^
Stage 4	Symptomatic cardiovascular disease in individuals with obesity and other risk factors for MS or CKD	Treatment of the disease and clinical manifestations	
4a	No renal failure		
4b	Renal failure present		eGFR < 29 mL/min/1.73 m^2^ and/or Albuminuria ≥ 30 mg/g

BMI, body mass index; HDLC, high-density lipoprotein cholesterol; NT-proBNP, N-terminal pro b-type natriuretic peptide; HF, heart failure; CKD, chronic kidney disease; eGFR, estimated glomerular filtration rate; MS, metabolic syndrome.

**Table 4:** Review of diagnostic methods used in the clinical assessment of the cardiovascular-kidney-metabolic syndrome.

Dis.	Screening Test	Advantages	Disadvantages
Kidney diseases	eGFR	Allows for the assessment of chronic kidney disease. Takes into account age and sex.	The test is insensitive in the early stages of the disease. Depends on muscle mass.
	albuminuria	Early marker of kidney damage. Reasonably accessible test. Requires no patient preparation.	It can increase, for example, after exercise, fever, pregnancy, infection, significant hyperglycemia, hypertension, urinary tract infection, and hematuria. Quite expensive. Due to the high variability in urine excretion, it is recommended to perform two tests within 3 to 6 months. Urine from the first morning urine sample, midstream.
	creatinine	Widely available and inexpensive testing. Greater specificity and reproducibility of enzymatic methods.	Low sensitivity in the early stages of the disease. Interferes with the Jaffe method (e.g., bilirubin, glucose, medications, ketone bodies—enzymatic testing recommended). High cost of enzymatic methods. Depends on muscle mass. Schwartz’s GFR assessment may lead to overestimation.
	cystatin C	Concentration is independent of the patient’s age, sex, race, body weight, and hydration status.	Expensive testing. Low availability of testing in medical laboratories.
	urinalysis *	Fast. Inexpensive. Non-invasive. Easily accessible testing.	Low specificity. Limited sensitivity. Presence of false positive and negative results.
Diabetes	fasting glucose	Rapid screening test. Inexpensive. Widely available. Standardized method. Short turnaround time.	Low sensitivity in detecting early stages of diabetes. Sample stability (use of dedicated anticoagulants required). If NaF anticoagulants are not used, rapid centrifugation and testing are necessary. Preparation for testing is required (fasting).
	HbA1c	Reflects average glucose levels over the last 3 months. No preparation required. Standardized method.	Anemia, blood transfusions, and hemolytic diseases limit the use of this test. Other forms of hemoglobin, such as S, C, D, and E, may affect test results. This test is quite expensive.
	glucose tolerance test	High sensitivity. Inexpensive. Widely available.	The examination is time-consuming and burdensome for the patient.
Cardiovascular diseases	Lipidogram	Easily accessible test. Affordable. Multi-parameter. No preparation required.	Does not take into account the size of LDL particles.
	troponin T	A highly sensitive and specific marker of myocardial infarction. Early detection (4–8 h after infarction). Prognostic value. Sample stability. Result standardization.	Expensive test. It is not specific to heart attack (e.g., increases in heart failure, pulmonary embolism, myocarditis, kidney failure, and intense physical activity).
	NT-proBNP	Early diagnosis of heart failure. Assessment of disease severity. Monitoring therapy. Prognostic value. Distinguishing the causes of dyspnea.	Age- and sex-dependent: increases in older adults and women. Increases in patients with renal failure. Expensive test. Non-specific (may increase in conditions such as lung disease, sepsis, and chronic kidney disease).
MS	fasting glu.	described above	described above
	Triglycerides	Affordable testing. Short turnaround time for results.	Interferences may overestimate or underestimate the results (e.g., ascorbic acid, dicynone, metamizole).
	HDLC	Inexpensive test. Short turnaround time. Standardized method.	Interferences can overestimate/underestimate results; for example, elevated free fatty acid and denatured protein levels can increase HDLC levels. Impaired liver function—results may be unreliable.

Dis., disease entity or syndrome; * urine sediment: urine hematuria and red cell casts; HDLC, high-density lipoprotein cholesterol; (e)GFR, (estimated) glomerular filtration rate; LDL, low-density lipoprotein; NT-proBNP, N-terminal pro b-type natriuretic peptide; MS, metabolic syndrome; glu., glucose.

**Table 5:** Comparison of guidelines for CKD diagnosis based on current KDIGO (Kidney Disease: Improving Global Outcomes), ESC/EASD (European Society of Cardiology/European Association for the Study of Diabetes), and ADA (American Diabetes Association) indications for the diagnosis and approach to the cardiovascular-kidney-metabolic syndrome.

	KDIGO	ESC/EASD	ADA	General Meaning for CKMS
Scope of the Guidelines	Kidney disease, especially chronic kidney disease (CKD)	Cardiovascular disease (CVD) in people with diabetes	Comprehensive diabetes care, including cardiac and renal complications	Three perspectives: renal, cardiac, and metabolic—the foundation of CKMS
Definition of CKD	CKD = eGFR < 60 mL/min/1.73 m^2^ ≥ 3 months or albuminuria (ACR) ≥ 30 mg/g.	Adopts the KDIGO definition.	Adopts the KDIGO definition.	A unified definition allows for common CKMS assessment algorithms.
Classification of CKD	Two-dimensional: glomerular filtration rate (eGFR, G1–G5) + albuminuria (ACR, A1–A3).	Uses the KDIGO classification to assess cardiovascular (CV) risk.	Uses the KDIGO classification to assess diabetes complications.	The G/A grid is the basis for renal risk assessment in the CKMS.
Screening CKD in People with Diabetes	Annually eGFR + ACR in type 2 diabetes (T2D) and type 1 diabetes (T1D) > 5 years	Recommends renal assessment as part of CV risk assessment.	Annually: eGFR + ACR in all patients with diabetes.	Standard approach: CKD is treated as a key component of CKMS.
Monitoring CKD	Frequency depends on the G/A category (from 1/year to 4/year)	Emphasizes the need for monitoring eGFR and ACR in high-risk patients.	Monitoring depends on the stage of CKD and the presence of albuminuria.	Regular renal monitoring is the basis for assessing CKMS progression.
Albuminuria—Importance	A key marker of kidney damage and CV risk.	A strong predictor of CV events.	One of the leading indicators of diabetes complications.	Albuminuria = a common biomarker of CKMS.
Cardiovascular (CV) Risk Assessment	CKD = high CV risk equivalent	Uses SCORE2 Diabetes and risk classes (high/very high)	Risk based on presence of CKD, HF, ASCVD (Atherosclerotic Cardiovascular Disease)	Integration: CKD automatically increases CV risk → key in CKMS.
Cardiac Function Assessment	Recommends the evaluation of HF (Heart Failure) in patients with CKD (e.g., NT proBNP, echo)	Detailed diagnostic algorithms for HF in people with diabetes.	Recommends the evaluation of HF as a complication of diabetes and CKD.	HF is one of the three pillars of CKMS.
Metabolic parameters	Emphasizes the importance of metabolic control.	Highlights the role of obesity, dyslipidemia, and insulin resistance in CV risk.	Specific targets for glycemia, lipids, and body weight.	The metabolic component is key to CKM.
Lifestyle	Recommends sodium reduction, weight control, and physical activity.	Emphasis on diet, activity, and weight loss.	Strong focus on diet, activity, and weight loss.	Lifestyle is the everyday basis for CKM prevention.
Early detection of complications	CKD as a “silent disease” → emphasis on screening.	Early identification of CVD in people with diabetes.	Early detection of CKD, CVD, and neuropathy.	Early diagnosis is the key to stopping the progression of CKD.
Multidisciplinary approach	Collaboration between a nephrologist, a diabetologist, and a cardiologist	Collaboration between a diabetologist and a cardiologist	Collaboration between a diabetologist, a nephrologist, and a cardiologist	CKM care model = integration of three specializations

## Data Availability

No new data were created or analyzed in this study. Data sharing is not applicable to this article.

## Abbreviations

The following abbreviations are used in this manuscript:

AACE	American Association of Clinical Endocrinology
ADA	American Diabetes Association
AGEs	glycation end-products
AHA	American Heart Association
BMI	body mass index
C	cholesterol
CKD	chronic kidney disease
CKMS	cardiovascular-kidney-metabolic syndrome
creat	creatinine
CVD	cardiovascular disease
CysC	cystatin C
Dis.	disease entity or syndrome
eGFR	estimated glomerular filtration rate
EGIR	European Group for the Study of Insulin Resistance
eNOS	decreased endothelial nitric oxide synthase
ESC	European Society of Cardiology
glu.	glucose;
HDL	high-density lipoprotein c
HF	heart failure
KDIGO	Kidney Disease: Improving Global Outcomes
LDL	low-density lipoprotein
MS	metabolic syndrome
NHLBI	National Heart, Lung, and Blood Institute
NT-proBNP	N-terminal pro B-type natriuretic peptide
OGTT	oral glucose tolerance test
PKC	protein kinase C
POLSPEN	Polish Society for Parenteral, Enteral Nutrition and Metabolism
PTLO	Polish Society for Obesity Treatment
PTMR	Polish Society of Family Medicine
PTNT	Polish Society of Hypertension
RAAS	renin-angiotensin-aldosterone system
ROS	reactive oxygen species
SDOH	Social Determinants of Health
SUA	serum uric acid
syn.	syndrome
WHO	World Health Organization
